# Solution Behavior of Glyco-Copoly(l-Glutamic Acid)s in Dilute Saline Solution

**DOI:** 10.1021/acs.biomac.4c00288

**Published:** 2024-05-14

**Authors:** Dimitrios Skoulas, Olusola Mary Ojo, Anja Thalhammer, Zdravko Kochovski, Helmut Schlaad

**Affiliations:** † Institute of Chemistry, University of Potsdam, Karl-Liebknecht-Str. 24-25, 14476 Potsdam, Germany; ‡ Institute of Biochemistry and Biology, Karl-Liebknecht-Str. 24-25, 14476 Potsdam, Germany; § Institute for Electrochemical Energy Storage, Helmholtz-Zentrum Berlin, Hahn-Meitner Platz 1, 14109 Berlin, Germany

## Abstract

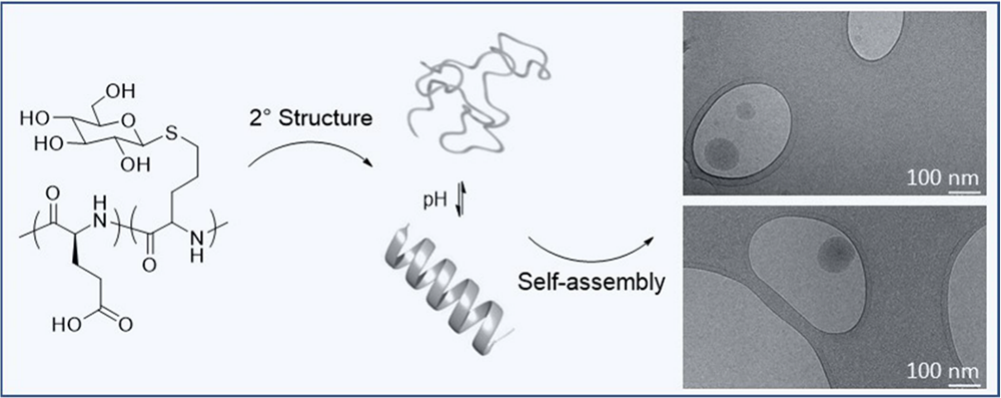

A small series of copoly(α,l-glutamic acid/dl-allylglycine)s with the same chain length and allylglycine content (∼10 mol %) but different spatial distribution of allylglycine units was synthesized and subsequently glycosylated via thiol-ene chemistry. Dilute aqueous copolypeptide solutions (0.1 wt %, physiological saline) were analyzed by circular dichroism spectroscopy, dynamic light scattering, and cryogenic transmission electron microscopy. The copolypeptides adopted a random coil or α-helix conformation, depending on solution pH, and the glycosylated residues either distorted or enhanced the folding into an α-helix depending on their location and spatial distribution along the chain. However, regardless of their secondary structure and degree of charging, all partially glycosylated copolypeptides self-assembled into 3D spherical structures, supposedly driven by a hydrophilic effect promoting microphase separation into glucose-rich and glutamate-rich domains.

## Introduction

Synthetic glycopolymers contain mono- or oligosaccharide (glycan) moieties that are covalently attached to the polymer main chain and can therefore be regarded as simple models of natural glycoproteins or proteoglycans. Due to the importance of sugars for cell recognition, immune response, and other biological processes, glycopolymers are considered as promising smart functional materials for biomedical or life science applications. A plethora of linear, cyclic, and branched glycopolymers as well as (multi)block and sequence-defined glycopolymers has been synthesized aiming at amphiphilic or stimuli-responsive architectures mostly for targeted drug delivery and lectin-binding studies.^1,2^

However, most of these sophisticated glycopolymer architectures are far off the chemical structure of natural glycoproteins, which are linear protein chains to which glycans are usually directly *N*-linked to asparagine or *O*-linked to serine or threonine residues, and their high complexity and biological function arises from the protein primary structure and the varying amount and diverse chemical structures of attached glycans (and protein secondary and tertiary structure).^3^ The most simple mimic of a glycoprotein would however be a (partially) glycosylated linear homopolypeptide, which can be synthesized by ring-opening polymerization of amino acid *N*-carboxyanhydrides (NCAs), either by direct (co)polymerization of glycosylated monomers or by glycosylation of preformed polypeptides.^4−6^ Such synthetic glyco(co)polypeptides would not have a specific amino acid sequence and randomly placed glycosylated residues, nevertheless they are well able to fold into distinct secondary structures, preferably α-helices (however, β-sheets in glycopolypeptides have not been observed yet),^7^ and interact with lectins.^6^

Poly(glyco-l-lysine)s^8^ and poly(glyco-l-(homo)cysteine)s^9^ were reported to preferably adopt an α-helical conformation in aqueous media. The sugars did not interfere with the conformation of the polypeptides when positioned far enough away from the backbone, while polar (sulfone) linker groups in close proximity to the backbone were able to disrupt the α-helix. Partially glycosylated poly(l-glutamic acid)s with a degree of glycosylation of 0.1–0.8 showed a coil-to-helix transition in aqueous media in dependence of solution pH.^10−13^ The maximum helicity of the glycol-poly(l-glutamic acid)s, which were prepared by amide coupling of glucosamine to poly(l-glutamic acid), was found to decrease with increasing sugar density, and the close to 80% glycosylated sample adopted a random coil rather than an α-helical conformation. The destabilization of the glycosylated helix was explained with an increased steric repulsion due to the binding of water molecule clusters to the hydrophilic sugar moieties,^10^ although it could have also originated from competing hydrogen bonding interactions of the short amide linker side chains with the polypeptide backbone.^14^ Partially glycosylated poly(l-glutamic acid)s, in which the sugars were covalently attached to the polypeptide via nonpolar thioether or longer hydrophobic amide linkers, exhibited an α-helical conformation, as expected, and the glycosylated side chains seemed to stabilize rather than destabilize the helix.^11−13^

The effect of glycosylation of polypeptide chains on their secondary structure is not fully understood yet. Maybe the sugar itself plays a minor role and it is mainly the linker group that determines the conformation of the polypeptide chain. However, this idea of binding water molecule clusters to the hydrophilic sugar moieties suggests that glycopolypeptides could possibly self-assemble into aggregates in an aqueous solution driven by a preferential hydration of the sugars or a hydrophilic effect. This effect is related to the separation of two water-soluble polymers into two aqueous phases (aqueous two-phase system, ATPS)^15^ and the aqueous self-assembly of double-hydrophilic block copolymers^16^ into vesicles or giant vesicles.^17,18^

In this work, we synthesized a small series of copoly(α,l-glutamic acid/dl-allylglcyine)s having the same chain length and composition (∼10 mol % allylglycine) but differing in spatial distribution of allylglycine units (from statistical to gradient). Allylglycine residues were afterward used to attach 1-thio-glucose via thiol-ene chemistry. The nonglycosylated and glycosylated copolypeptides were analyzed in dilute physiological saline solution at different pH, applying circular dichroism (CD) spectroscopy, dynamic light scattering (DLS), and cryogenic transmission electron microscopy (cryo-TEM) to elucidate the impact of glycosylation on the secondary structure and the self-assembly behavior.

## Experimental Section

### Chemicals and Materials

γ-Benzyl l-glutamate (BLG, ≥ 99%) was purchased from Acros Organics. Bis(trichloromethyl) carbonate (triphosgene, > 99%) was purchased from Fluorochem. dl-Allylglycine (AG, > 98%) and neopentyl amine (>98%) were purchased from TCI. α-Pinene (≥99%), trifluoroacetic acid (TFA, 99%), methanesulfonic acid (MSA, ≥ 99%), anisole (99%), 1-thio-β-d-glucose sodium salt (98%), Dowex 50WX2 ion-exchange resin, 4-(2-hydroxyethoxy)-phenyl 2-hydroxy-2-propyl ketone (Irgacure 2959, 98%), acetic anhydride (99%), acetic acid (99.7%), sodium acetate (≥99%), sodium hydrogen carbonate (≥99.5%), sodium chloride (≥99%), hydrochloric acid (37%), sodium hydroxide (98%), *N,N*-dimethylformamide (DMF, 99.8%, anhydrous), tetrahydrofuran (THF, 99.8%, anhydrous), *n*-hexane (98%), ethyl acetate (99.8%, anhydrous), and diethyl ether (99%) were supplied by Sigma-Aldrich. Silica gel (high-purity grade) came from Fluka and was dried at 150 °C for 48 h. Dialysis membranes (regenerated cellulose, MWCO 1000 Da) were received from Fisher Scientific. Deuterated solvents, CDCl_3_ (99.8% D), DMSO-*d*_6_ (99.8% D), and D_2_O (99.9% D), were purchased from Deutero GmbH.

The *N*-carboxyanhydrides (NCAs) γ-benzyl l-glutamate (BLG) and dl-allylglycine (AG) were synthesized from BLG and AG, respectively, and triphosgene (2 equiv. with respect to amino acid) in dry THF at 70 °C under reflux; α-pinene was used to trap evolving HCl.^12^ The reaction mixture was stirred for 1 h (clearing of the solution occurred after about 30 min) and then bubbled with argon to remove excess triphosgene. The crude NCAs were recrystallized three times from ethyl acetate/hexane (1:4 v/v), filtered through a column filled with silica gel, evaporated, and dried in a vacuum at 50 °C for 24 h.

All polymerizations were performed in dry DMF solution under vacuum at room temperature using a dry Schlenk tube equipped with a magnetic stirrer.

Poly(γ-benzyl l-glutamate) (PBLG). A solution of 9.3 mg (0.107 mmol) neopentyl amine in 2 mL of DMF was added to 2.809 g (10.67 mmol) BLG-NCA in 8.7 mL of DMF. The mixture was stirred for 24 h (aliquots were taken at predetermined reaction times and analyzed by FT-IR spectroscopy for determination of NCA conversion, see Figure S1) and then quenched with acetic anhydride. The PBLG was precipitated thrice with diethyl ether, centrifuged, filtered, and dried in a vacuum at 50 °C.

Statistical copoly(γ-benzyl l-glutamate/allylglycine) (**1a**). A solution of 9.3 mg (0.107 mmol) neopentyl amine in 2 mL of DMF was added to a mixture of 2.809 g (10.67 mmol) BLG-NCA and 0.151 g (1.07 mmol) AG-NCA in 8.7 mL of DMF. The mixture was stirred for 24 h and then quenched with acetic anhydride. The copolypeptide was precipitated thrice with diethyl ether, centrifuged, filtered, and dried in a vacuum at 50 °C.

Gradient copoly(γ-benzyl l-glutamate/allylglycine) (**1b**–**c**). 0.151 g (1.07 mmol) AG-NCA in 2 mL of DMF after either 8 min (**1b**) or 50 min (**1c**) were added to stirred solutions of 2.809 g (10.67 mmol) BLG-NCA and 9.3 mg (0.107 mmol) neopentyl amine in 10.7 mL of DMF. The mixtures were stirred for another 24 h and then quenched with acetic anhydride. The copolypeptides were precipitated thrice with diethyl ether, centrifuged, filtered, and dried in a vacuum at 50 °C.

Poly(l-glutamic acid) (PLG) and copoly(l-glutamic acid/allylglycine) (**2a**–**c**). 1 g of benzyl-protected (co)polypeptide (∼4.5 mmol BLG units) was dissolved in 13.7 g (120 mmol) TFA. After cooling to 0 °C, 13.1 g (136 mmol) MSA and 2.16 g (20 mmol) anisole were added, and the mixture was stirred for 18 min at 0 °C and for another 20 min at room temperature. The debenzylated (co)polypeptides were precipitated into diethyl ether, centrifuged, redissolved in 10% aqueous NaHCO_3_, dialyzed (RC 1000) against water for 2 d, and freeze-dried. Yield: 500–700 mg.

1-Thio-β-d-glucose was prepared from the corresponding sodium salt: 1-thio-β- d-glucose sodium salt (1 g), Dowex 50WX2 (0.5 g), and water (10 mL) were mixed and stirred for 20 min. The homogeneous solution was filtered and freeze-dried overnight.

Glyco-copoly(l-glutamic acid) (**3a**–**c**). A solution of 0.5 g of copolypeptide **2a**–**c** (∼0.04 mmol allyl) in 5 mL of 0.1 M acetate buffer was degassed by bubbling with argon for 20 min. Then, 16.2 mg (0.08 mmol) 1-thio-β-d-glucose and 0.83 mg (3.7 μmol) Irgacure 2959 were added, and the mixture was irradiated with UV-A light (VL-215.BL, 2 × 15 W, λ = 365 nm) overnight while stirring vigorously. The mixture was then diluted with 20 mL of extra pure water and dialyzed (RC 1000) against water for 2 d. The glycosylated copoly(l-glutamic acid)s **3a**–**c** were isolated after freeze-drying from water. Yield: ∼ 500 mg.

### Analytical Instrumentation and Methods

Nuclear magnetic resonance (NMR) spectra were recorded on a Bruker AVANCE NEO 400 MHz spectrometer at room temperature. Samples were prepared in CDCl_3_, DMSO-*d*_6_, or D_2_O, and signals were referenced to the respective solvent peaks; CDCl_3_ δ (^1^H) 7.26 ppm, DMSO-*d*_6_ δ (^1^H) 2.50 ppm, D_2_O δ (^1^H) 4.65 ppm.

Fourier-transform infrared (FT-IR) spectra were recorded on a Bruker Vertex 70 fitted with a PLATINUM ATR. Samples were placed directly on the ATR diamond under an argon flow. Spectra were acquired and processed with the OPUS 7.0 software.

Size exclusion chromatography (SEC) with simultaneous UV and RI (differential refractive index) detection was performed with *N*-methyl-2-pyrrolidone (NMP) + 0.5 wt % LiBr as the eluent (flow rate: 0.5 mL min^–1^) at room temperature. The stationary phase was a 300 × 8 mm^2^ PSS-GRAM analytical linear column (particle size 7 μm, separation range 10^2^–10^6^ Da). Solutions containing ∼0.15 wt % polymer were filtered through 0.45 μm filters; the injected volume was 100 μL. Polystyrene standards (PSS, Mainz, Germany) were used for calibration.

CD spectroscopy was performed with a JASCO J-815 spectrometer (Jasco, Pfungstadt, Germany) at 20 °C. The instrument was calibrated with 1S-(+)-10-camphorsulfonic acid. For the analysis of 0.1 wt % polypeptide saline solution (0.9 wt % NaCl), a quartz cell with an optical path length of 0.1 mm was used. Spectra were recorded in the wavelength range of 190–250 nm with an integration duration of 8 s and corrected by subtraction of the respective background spectrum. Measured ellipticities θ (in units of degree) were converted into molar ellipticities [θ] (deg cm^2^ dmol^–1^) using the equation: [θ] = θ × 100 × M/(*c* × *l*), where *M* is the average molecular weight of residues in the polypeptide (g mol^–1^), *c* is the polypeptide concentration (mg mL^–1^), and *l* is the cell path length (cm). The helicity of the polypeptide chain was calculated from the molar ellipticity value at λ = 222 nm: % α-helix = (−[θ]_222_ + 3000)/39,000) × 100%.^19^

DLS was performed with a Malvern Zetasizer Nano ZS at room temperature. The 0.1 wt % polypeptide saline solutions were filtered through 0.45 μm filters into 1.5 mL disposable glass cuvettes.

Cryo-TEM was performed using a JEOL JEM-2100 transmission electron microscope (JEOL GmbH, Eching, Germany). Cryo-TEM specimens were prepared as follows: a 4 μL drop of sample dispersion was deposited on a lacey carbon-coated copper TEM grid (200 mesh, Electron Microscopy Sciences, Hatfield, PA), and then plunge-frozen in liquid ethane at its freezing point with a FEI vitrobot Mark IV (setting condition: 4 °C and 95% humidity). Vitrified grids were either transferred directly to the microscope cryo transfer holder (Gatan 914, Gatan, Munich, Germany) or stored in liquid nitrogen. All grids were glow-discharged before the experiment. Imaging was carried out at temperatures around 90 K. The TEM was operated at an acceleration voltage of 200 kV, and a defocus of the objective lens of about 1.5–2 μm was used to increase the contrast. Cryo-EM micrographs were recorded with a bottom-mounted 4 × 4k CMOS camera (TemCam-F416, TVIPS, Gauting, Germany) at a magnification of 50,000×, corresponding to a pixel size of 2.32 Å at the specimen level. The total electron dose in each micrograph was kept below 15 e^–^/Å^2^.

## Results and Discussion

### Synthesis of Statistical and Gradient Glyco-Copolypeptides

**Scheme 1:**
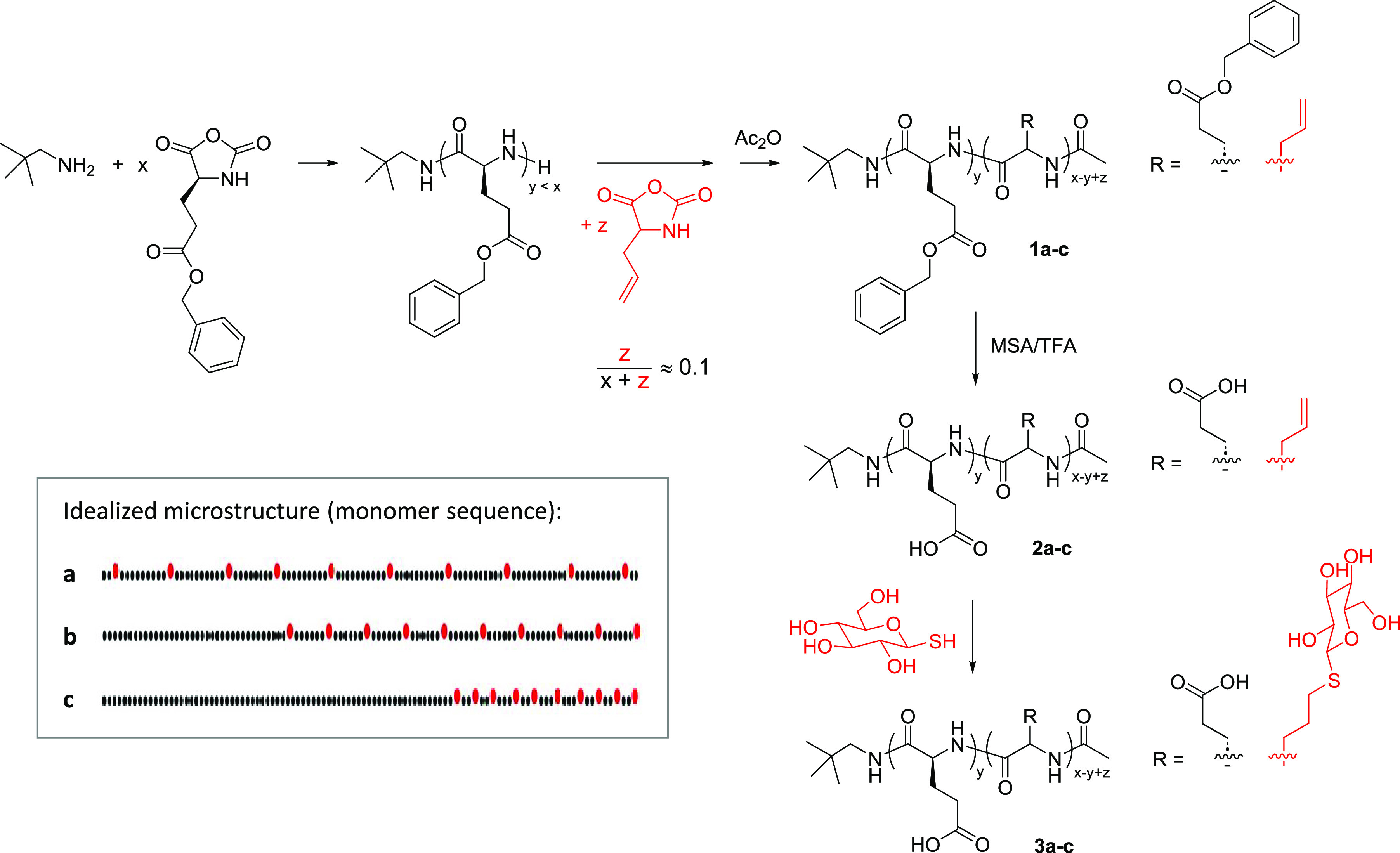
Synthetic Pathway toward Glycosylated Copoly(l-Glutamic Acid)s

A small series of glycosylated copoly(α,l-glutamic acid)s having the same average molar mass and composition (∼10 mol % glycosylated units) but different microstructures was synthesized as outlined in Scheme 1.^12^ The first step involved the ring-opening copolymerization of a mixture of γ-benzyl l-glutamate (BLG) NCA and dl-allylglycine (AG) NCA (100:13), initiated by neopentyl amine, in *N,N*-dimethylformamide (DMF) solution ([NCA]_0_ ≈ 1.1 M) at room temperature for 24 h to give a statistical poly(BLG-*co*-AG) **1a** (assuming the same or similar reactivity of the two NCAs).^12^ Starting the polymerization with pure BLG NCA and adding the AG NCA after a predetermined reaction time, either at 8 min (∼30% conversion) or 50 min (∼70% conversion, see Figure S1 in the Supporting Information), produced the gradient-like copolypeptides **1b** and **1c**, respectively, with a poly(BLG) (PBLG) first block and a statistical copoly(BLG/AG) second block (see the inset in Scheme 1). A PBLG homopolymer was also synthesized as reference material.

**Figure 1:**
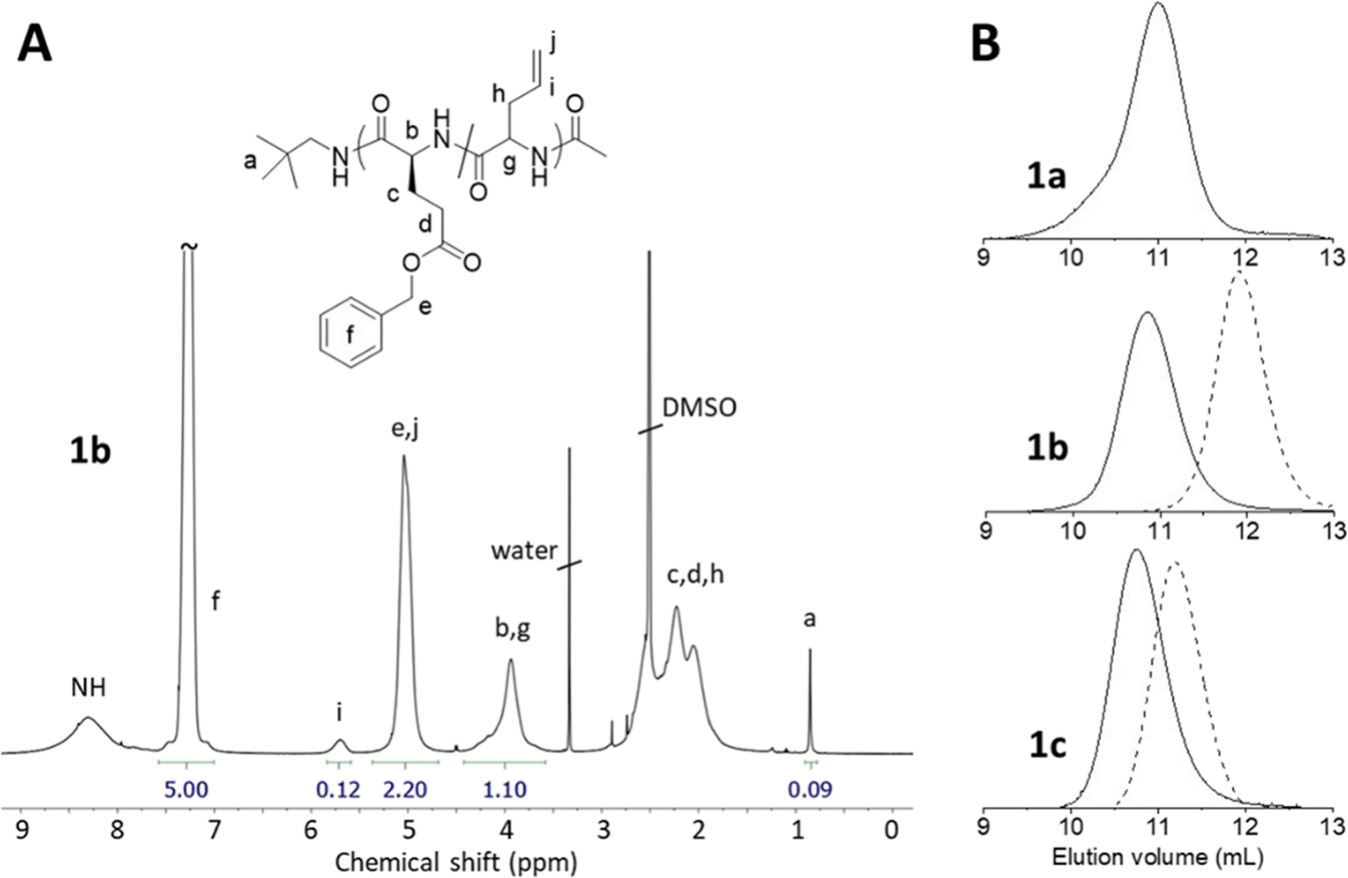
(A) Exemplary ^1^H NMR spectrum (400 MHz, DMSO-*d*_6_) of BLG/AG copolypeptide **1b**. (B) SEC-RI traces of copolypeptides **1a**–**c** (solid lines) and the corresponding PBLG first block precursors of **1b** and **1c** (dashed lines).

The copolypeptides were characterized by ^1^H NMR spectroscopy and SEC (see Figure 1 and Supporting Information). Results are summarized in Table 1. SEC suggested that the three copolypeptides **1a**–**c** have the same apparent molar mass (*M*_n_^app^ ∼ 14 kg mol^–1^) and low dispersity (*Đ* < 1.2). According to ^1^H NMR analysis, **1a**–**c** exhibit the same (absolute) number–average degree of polymerization, DP = 110, which equals the targeted DP at quantitative NCA conversion, and the same overall composition, mole fraction of AG *x*_AG_ = 0.11–0.12, which equals *x*_AG_ in the monomer feed. The PBLG first block (which was taken from the reaction mixture prior to the addition of AG) of **1b** was found to contain 29 BLG units and that of **1c** 68 BLG units. Accordingly, the mole fraction of AG in the second copoly(BLG/AG) segment increases from 0.12 (**1a**) to 0.15 (**1b**) to 0.31 (**1c**) and the average spacing between AG units tentatively decreases from about 8 to 7 to 3, respectively, as illustrated in Scheme 1.

**Table 1:** Molecular Characteristics of PBLG and BLG/AG Copolypeptides **1a**–**c** Obtained by Neopentyl Amine-Initiated Ring-Opening Polymerization of BLG/AG NCA ([Amine]_0_:[BLG NCA]_0_:[AG NCA]_0_ = 1:100:10) at Room Temperature for 24 h

	*M*_n_^app^ (kg mol^–1^)^a^	*Đ*^a^	*x*_AG_^b^	*DP*^c^	*x*_helix_^e^ (%)
PBLG	13.1	1.13		102	77
**1a**	14.1	1.18	0.12	110	73
**1b**	14.3	1.13	0.11	110 (29)^d^	66 (65)^f^
**1c**	14.8	1.17	0.12	110 (68)^d^	65 (78)^f^

a Apparent number-average molar mass (*M*_n_^app^) and dispersity (*Đ*) determined by SEC (eluent: NMP, polystyrene calibration).

b Mole fraction of allylglycine in the copolypeptide by ^1^H NMR spectroscopy (ratio of peak integrals (*i*)/(*b* + *g*); see Figure 1).

c Number-average degree of polymerization by ^1^H NMR spectroscopy (ratio of peak integrals (*b* + *g*)/(*a*/9*)*, see Figure 1).

d Number-average degree of polymerization of the PBLG first block precursor.

e %Helical conformation by ^1^H NMR spectroscopy (DMSO-*d*_6_, αCH (*b*, *g*): δ 3.80–4.05 ppm, right-handed α-helix, δ 4.05–4.60 ppm, random coil, according to ref (20)).

f %Helical conformation of the PBLG first block precursor.

In the second step, the copolypeptides **1a**–**c** and PBLG were treated with methanesulfonic acid (MSA) and anisole in TFA at 0 °C for the debenzylation of BLG units, thereby avoiding racemization of the polypeptides, to give the copoly(l-glutamic acid/allylglycine)s **2a**–**c** and PLG, respectively. ^1^H NMR analysis indicated for all samples quantitative debenzylation (>99%) (considering the signal *f* of residual phenyl groups) and virtually no change in DP (ratio of signal intensities (*b* + *g*)/(*a*/9)) (see the ^1^H NMR spectra in Figure S4).

Finally, the allylglycine units of the debenzylated copolypeptides **2a**–**c** were glycosylated with 1-thio-β-d-glucose in 0.1 M acetate buffer solution using 4-(2-hydroxyethoxy)-phenyl 2-hydroxy-2-propyl ketone (Irgacure 2959) as the photoinitiator and irradiation with UVA light (λ = 365 nm) for 24 h at room temperature. ^1^H NMR analysis of the obtained copolypeptides **3a**–**c** showed the presence of glucose units (δ 3.0–4.0, 4.5 ppm)^12^ but also of unreacted allyl groups (∼15 to 18%, by comparing the ratio of signal intensities *i*/*a* of **3a**–**c** and **2a**–**c**) (see the ^1^H NMR spectra in Figure S5 and Table 2), which however was earlier not observed for copoly(l-glutamic acid/allylglycine) of DP ∼ 50 (complete disappearance of allyl groups).^12^ Notably, a second glycosylation of the partially glycosylated samples **3a**–**c** did not improve the degree of glycosylation beyond 85%. It is hypothesized that these unreacted allyl groups were however not accessible by thiyl radicals, possibly due to steric hindrance, thus AG units might not be evenly distributed along the copolypeptide chain, as illustrated in Scheme 1, and shielded by glycosylated side chains in too close proximity.

**Table 2:** Results of Debenzylation of PBLG and Copolypeptides **1a**–**c** with MSA/TFA, to Give PLG and the Copoly(l-glutamic Acid/Allylglycine)s **2a**–**c**, and Glycosylation of **2a**–**c** with 1-Thio-β-d-glucose, to Give the Glycosylated Copoly(l-Glutamic Acid)s **3a**–**c**, Respectively

precursor		DD^a^ (%)		DG^b^ (%)	*x*_glyco_^c^
PBLG	PLG	>99			
**1a**	**2a**	>99	**3a**	80	0.10
**1b**	**2b**	>99	**3b**	85	0.09
**1c**	**2c**	>99	**3c**	82	0.10

a Degree of debenzylation determined by ^1^H NMR spectroscopy (peak integral (*a*) ≡ 0.09H, DD = 1-peak integral (*f*)/5H.

b Degree of glycosylation determined by ^1^H NMR spectroscopy (peak integral (*a*) ≡ 0.09H, DG = 1-peak integral (*i*, **3a**–**c**)/(*i*, **2a**–**c**)).

c Mole fraction of glycosylated AG units, *x*_glyco_ = DG·*x*_AG_.

### Secondary Structure

The ^1^H NMR signal of the αCH proton (δ ∼ 4 ppm) of PBLG and the copolypeptides **1a**–**c** was used to gain information about the conformation or secondary structure of the polypeptide chains in DMSO-*d*_6_ solution, referring to the detailed NMR studies by Bradbury et al.^20^ Resonances of the αCH proton appearing at δ 3.80–4.05 and 4.05–4.45 ppm (see Figures S2 and S3) were assigned to peptide units in right-handed α-helix and random coil conformations, respectively. Accordingly, PBLG (DP 102) and PBLG (DP 68) exhibited a close to 80% α-helical conformation, which is however less than the expected full helical conformation of high molar mass PBLG.^20^ (Notably, DMSO is regarded as a weaker helix-supporter as compared to, for instance, chloroform^20^ and the PBLG (DP 102) adopted a nearly perfect helical conformation in CDCl_3_ solution, see Figure S2). For the shorter PBLG (DP 29), the helicity further decreased to 65% (Table 1), as expected. The helicities of the copolypeptides **1a**–**c** (DP 110) were about 70%, thus lower as for PBLG (DP 102), which could be attributed to the presence of dl-allylglycine units disturbing the formation of the α-helix.^13^ The effect of AG units on the conformation was most pronounced for **1c** having a strong gradient-like microstructure. The helicity of **1c** was found to be just 65% while it was 78% for its PBLG (DP 68) first block precursor. Accordingly, the statistical BLG/AG second block (DP 42, mole fraction AG 0.31) had a calculated helicity of just ∼44% and thus was predominantly in a random coil conformation.

The secondary structures of the water-soluble PLG and the copolypeptides **2a**–**c** and **3a**–**c** in dependence on pH were analyzed by CD spectroscopy. Samples containing 0.1 wt % polypeptide in physiological saline solution (0.9 wt % NaCl) were titrated with 0.1 M aqueous HCl or NaOH to adjust the pH value; the pH was measured with a pH meter prior to CD measurement. (Note: due to the relatively high concentration of the samples, CD measurements were conducted with a cuvette path length of 0.1 mm).

**Figure 2:**
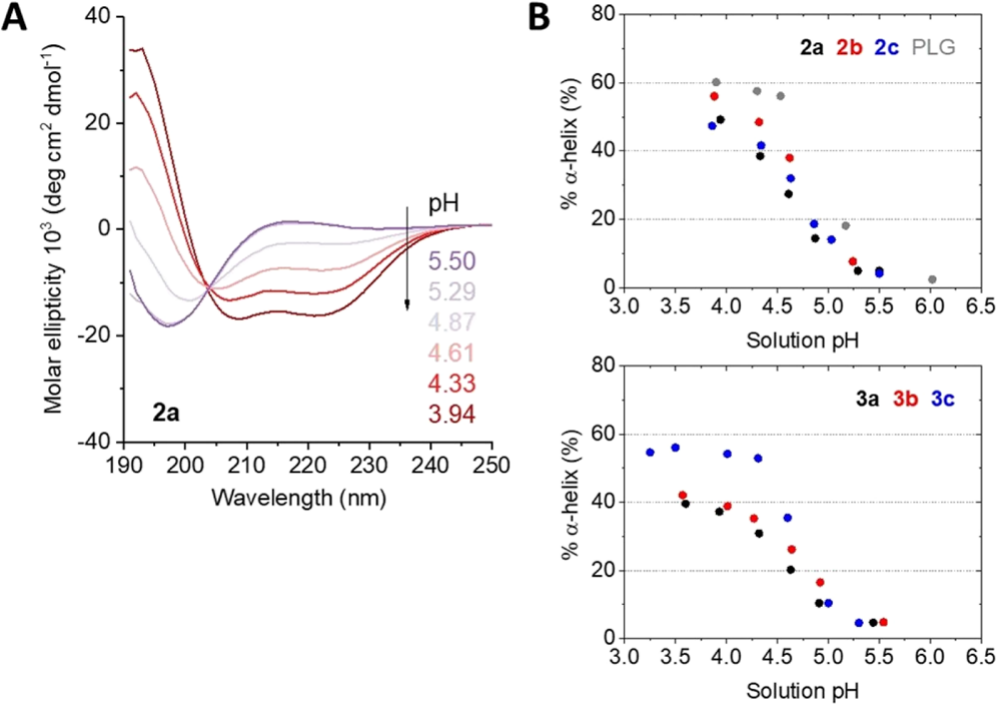
(A) Exemplary CD spectra of 0.1 wt % physiological saline solution of **2a** at different pH values at room temperature. (B) Dependence of helicity on solution pH for PLG and copoly(l-glutamic acid/allylglycine) **2a**–**c** (top) and glycosylated copoly(l-glutamic acid)s **3a**–**c** (bottom).

All samples showed the typical CD spectrum for polypeptides in random coil conformation at pH > 5 (that is when carboxyl side chains are deprotonated and charged, −COO^–^Na^+^) with a single minimum at λ = 196 nm. The transition to an α-helix conformation, as indicated by two characteristic minima at λ = 208 and 222 nm, started at around pH 5 reaching maximum helicity below pH 4 (when carboxyl side chains are protonated, −COOH) (see Figures 2 and S6). The appearance of an isodichroistic point at λ = 204 nm indicates that all polypeptide chains exclusively adopt random coil or α-helix conformations, thus excluding the presence of other conformations such as β-sheet.^21^ Notably, PLG in an aqueous solution, containing no extra added salt, showed the transition from random coil to α-helix at higher pH around 6.^13^

The pH-dependent helicity of the polypeptide chain was calculated from the molar ellipticity value at λ = 222 nm (see Experimental Section); results are shown in Figure 2B. The PLG reached a maximum helicity of ∼60% in saline solution at pH 3.9, which is lower than the ∼80% helicity of the PBLG precursor in DMSO solution. The decreased helicity of PLG in polar protic medium could be rationalized by competing hydrogen bonding interactions destabilizing the intramolecular hydrogen bonds in the helix (and the absence of π–π interactions, which additionally contribute to the stabilization of a PBLG helix).^22^ In the same line, the copolypeptides **2a**–**c** showed a similarly decreased helicity down to ∼50% at pH 3.9 as compared to **1a**–**c** (∼70% helicity in DMSO). Below pH 3.9, neither sample was stable and started to precipitate out of solution. However, samples seemed to be better soluble in saline solution than in pure water in which precipitation started to happen at around pH 4.7.^13^

The glycosylated copolypeptides **3a**–**c** remained in solution down to pH 3.5 or even pH 3.3 (**3c**). The maximum helicity of **3a** and **3b** was just ∼40%, which was lower than that of the corresponding nonglycosylated precursors **2a** (56%) and **2b** (49%), whereas **3c** reached a maximum helicity of 56% higher than that of **2c** (47%) and close to the 60% of the PLG reference sample. Evidently, the effect of the glycosylated residues on the secondary structure of the copolypeptides is different depending on the spatial distribution of glycosylated residues along the chain. Folding of the copolypeptide chain into an α-helix seemed to be disturbed for the statistical copolypeptide **3a**, and also **3b** in which on average every seventh to eighth unit is a glycosylated residue (see above). In line with this finding, it has been reported that the glycosylation of model proteins can result in local secondary structure distortion of α-helices^23^ and perturb protein folding (most prominently in the center of α-helices).^24^ However, earlier we observed just the opposite effect for a glycosylated statistical copoly(l-glutamic acid/dl-allylglycine) (DP 50, *x*_AG_ = 0.1) (measured at ∼0.02 wt % in water), which was comparable to **3a** albeit shorter in length.^12,13^ The gradient-like copolypeptide **3c**, on the other hand, contained the longest nonglycosylated α-helical PLG segment (DP 68) and glycosylated residues were locally separated and cumulated in the last third of the chain (about every third unit in average being a glycosylated residue, see above). Assuming that the PLG segment retained its secondary structure, the increased helicity of **3c** might have originated from an enhanced folding of the densely glycosylated segment from a random coil (see above) into a more α-helical conformation, possibly due to steric reasons and/or hydrogen bonding interactions between glucose and carboxylic acid side groups.^12^

### Self-Assembly

The 0.1 wt % polypeptide aqueous saline solutions were adjusted to around pH 4.3 (helical conformation) and pH 7.1 (random coil conformation) and analyzed with DLS and cryo-TEM to gain information about the possible formation of aggregates and their morphology.

**Figure 3:**
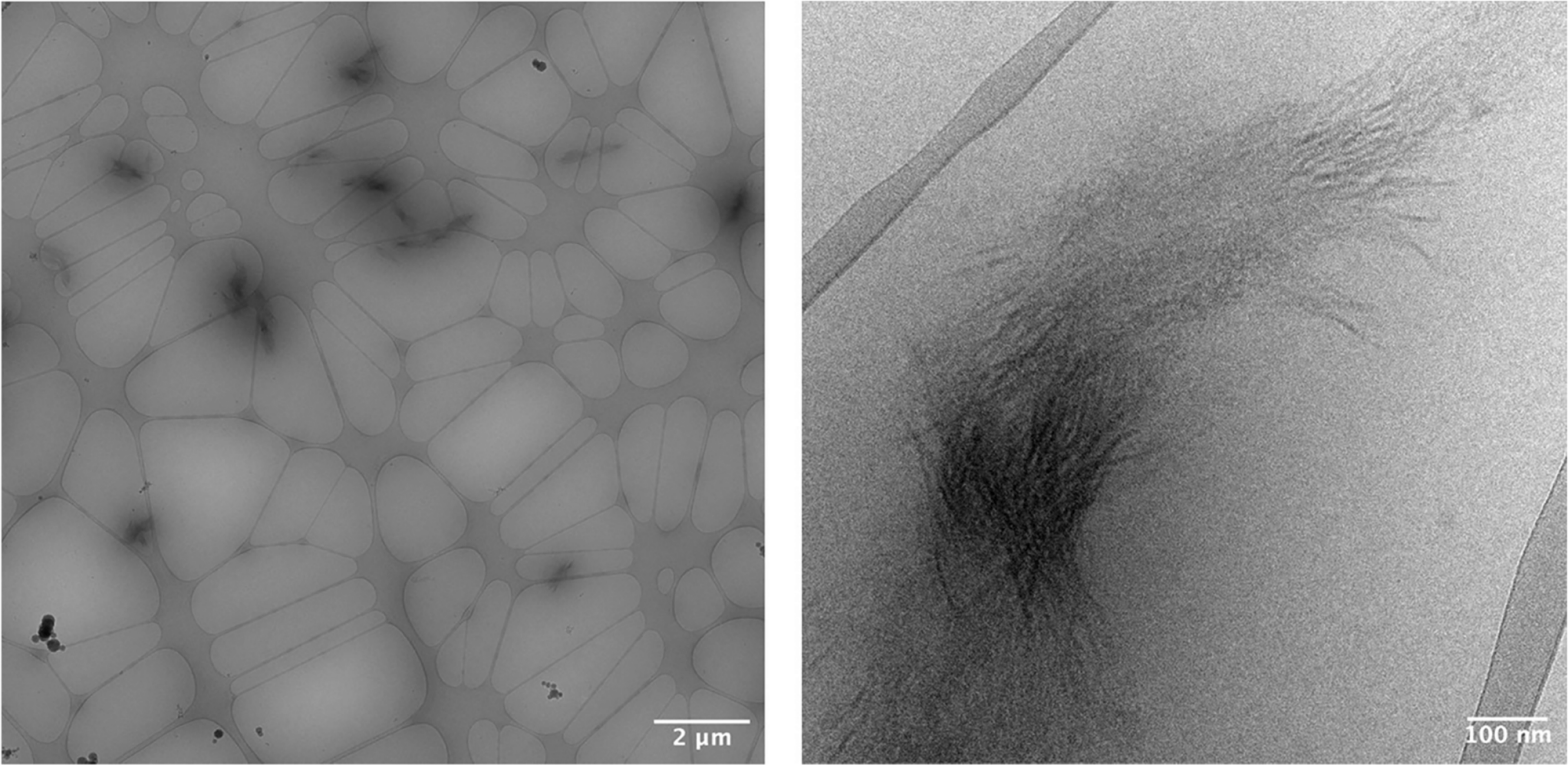
Cryo-TEM images of the aggregates formed in 0.1 wt % saline solution of copoly(l-glutamic acid/allylglycine) **2c** at pH 4.21; scale bars: 2 μm (left) and 100 nm (right).

DLS suggested that PLG and the copoly(l-glutamic acid/allylglycine)s **2a**–**c** did not form any kind of aggregates (particle diameter <10 nm, see Figure S7) at pH 7.1, that is when the copolypeptide chains are in random coil conformation and carboxylate side chains are fully charged (p*K*_a_ ≈ 4.3).^12^ The mole fraction of hydrophobic AG units of **2a**–**c** seemed to be too low, as expected, to induce the formation of stable aggregates. At around pH 4.3, still no aggregates could be observed for PLG and **2a**–**b**, while **2c** assembled into aggregates with an apparent diameter of ∼100 nm (see Figure S7). Cryo-TEM revealed the presence of large irregular aggregates or agglomerates of nanofibers, as shown in Figure 3. The formation of nanofibers might be rationalized considering an amphiphilic helical structure of **2c** chains, which consist of a partially deprotonated helical PLG segment (DP 68) and a less hydrophilic poly(l-glutamic acid/allylglycine) (DP 42, *x*_AG_ = 0.31) segment.

The situation changed completely for the glycosylated copoly(l-glutamic acid)s **3a**–**c** (glucose content ∼10% by weight), which all assembled into aggregates with a diameter of ∼100 nm (DLS, Figure S7) at either pH 7.1 or pH 4.4. The presence of aggregates at higher pH is especially surprising since the glyco-copolypeptide chains are fully charged and do not contain hydrophobic moieties (unlike the corresponding precursors **2a**–**c**, for which no aggregates were observed). We therefore hypothesized that the aggregation occurred possibly via attractive hydrogen bonding interactions between the glucose moieties and/or via a hydrophilic effect, rather than the conventional hydrophobic effect, as was earlier proposed for double-hydrophilic block copolymers.^17,18^ Binding of more water molecules to glucose than to carboxylate might create an osmotic pressure which is balanced out by microphase separation into an aqueous two-phase system (ATPS) of glucose-rich and glutamate-rich domains.^18^ The hydrophilic contrast between glucose and carboxylate should however increase with decreasing pH because the carboxylates become partially protonated and thus less hydrophilic.

**Figure 4:**
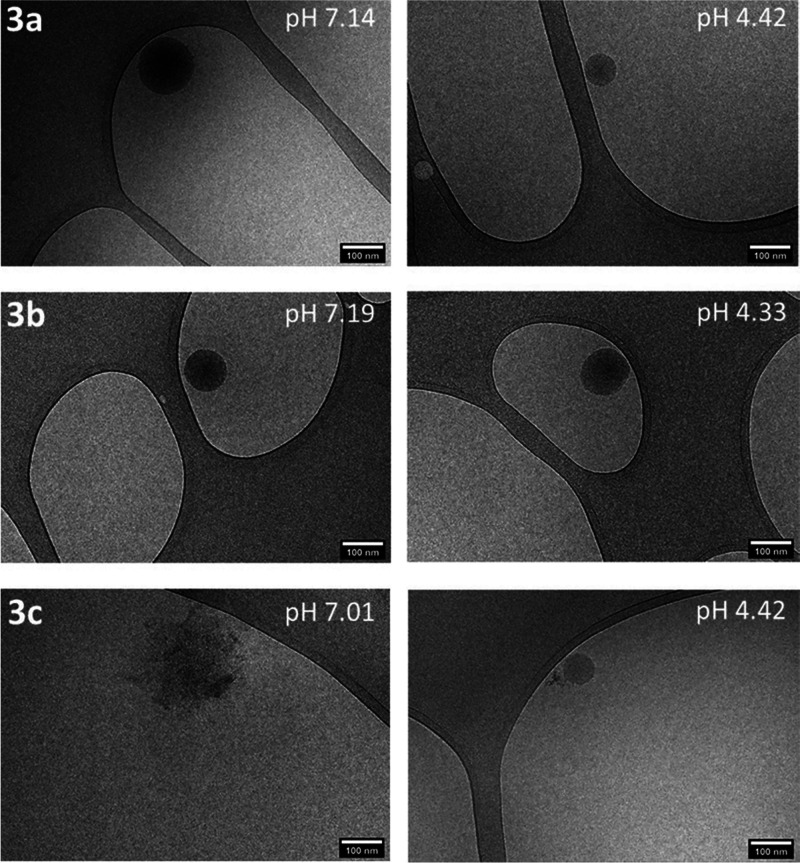
Cryo-TEM images of the aggregates formed in 0.1 wt % saline solution of glycosylated copoly(l-glutamic acid)s **3a**–**c** at around pH 7.1 (left) and pH 4.4 (right); scale bars: 100 nm.

The aggregates of **3a**–**c**, albeit low in number due to the high dilution of the samples, could be visualized by cryo-TEM (see Figure 4). Aggregates of **3a** and **3b** at around pH 7.2 were about 100 nm in diameter, which agreed reasonably well with the particle size determined by DLS (Figure S7), and were spherical in shape. For **3c** at pH 7.01, however, just irregular roundish structures could be observed. At lower pH 4.4, the assemblies of **3a**–**c** were all spherical in shape with a diameter of up to ∼100 nm; nanofibers, as seen for **2c** in an acidic environment (Figure 3), were not found. Albeit the spherical assemblies exhibited a cross-sectional constant low phase contrast against the vitreous ice, the line profiles indicated a higher electron density in their center (see Figure S8) and thus a 3D spherical rather than 2D disc-like structure. Also due to the low phase contrast, we could not reveal any inner structuring or two aqueous microphase-separated glucose-rich and glutamate-rich domains within the assemblies. Whether these assemblies are a kind of “large compound micelle”^25^ (notably, ordinary star-like micelles should be much smaller than ∼100 nm in size) or—as we tend to believe—vesicles cannot be judged at the moment.

## Conclusions

A series of three copoly(l-glutamic acid/dl-allylglcyine)s with the same chain length (DP 110) and composition (*x*_AG_ = 0.1) but different monomer sequence (from statistical to gradient-like) was synthesized and glycosylated with 1-thio-β-d-glucose. Nonglycosylated and glycosylated copolypeptides adopted a random coil conformation at neutral to basic pH and folded into an α-helix at acidic pH in saline solution. The gradient-like glyco-copoly(l-glutamic acid) reached a maximum helicity of 56% close to that of poly(l-glutamic acid) (60%), while that of the statistical copolypeptide was just ∼40% and even lower than that of the nonglycosylated precursor. Hence, glycosylated residues can distort or enhance the folding into an α-helix depending on their location and spatial distribution along the α-helical copolypeptide chain. However, regardless of their secondary structure and degree of charging, all glycosylated copoly(l-glutamic acid)s self-assembled into 3D spherical structures in dilute saline solution. Since the samples did not contain hydrophobic moieties, it is hypothesized that the formation of these copolypeptide assemblies originates from a hydrophilic effect promoting microphase separation into glucose-rich and glutamate-rich domains.

Future work will be devoted to a more detailed analysis of the structure of the aqueous glyco-copolypeptide assemblies and extended systematic study of the self-assembly behavior (variation of chain length, degree of glycosylation, sample concentration, solvent, etc.), possibly revealing not only the formation of spherical assemblies but also of glycosylated nanofibers or networks.^26,27^

## Supporting Information Available

Kinetics of BLG polymerization, ^1^H NMR spectra of all copolypeptide samples, CD spectra and DLS particle size distributions of 0.1 wt % copolypeptide saline solutions at different pH, and additional cryo-TEM images with electron density profiles (PDF)
