# Supplementary material for: Solution Behavior of Glyco-Copoly(l-Glutamic Acid)s in Dilute Saline Solution
Source: Biomacromolecules. 2024 May 14;25(6):3724–30. doi: 10.1021/acs.biomac.4c00288 (PMC11170941; doi:10.1021/acs.biomac.4c00288)
Supplement: Supplementary file 1 — bm4c00288_si_001.pdf [file bm4c00288_si_001.pdf]

Supporting Information

Solution behavior of glyco-copoly(L-glutamic acid)s in dilute saline solution

Dimitrios Skoulas[+], Olusola Mary Ojo[+], Anja Thalhammer[#], Zdravko Kochovski[§], and Helmut Schlaad[+]*

[+] Institute of Chemistry, University of Potsdam, Karl-Liebknecht-Str. 24-25, 14476 Potsdam, Germany. [#] Institute of Biochemistry and Biology, Karl-Liebknecht-Str. 24-25, 14476 Potsdam, Germany. [§] Institute for Electrochemical Energy Storage, Helmholtz-Zentrum Berlin, Hahn-Meitner Platz 1, 14109 Berlin, Germany.

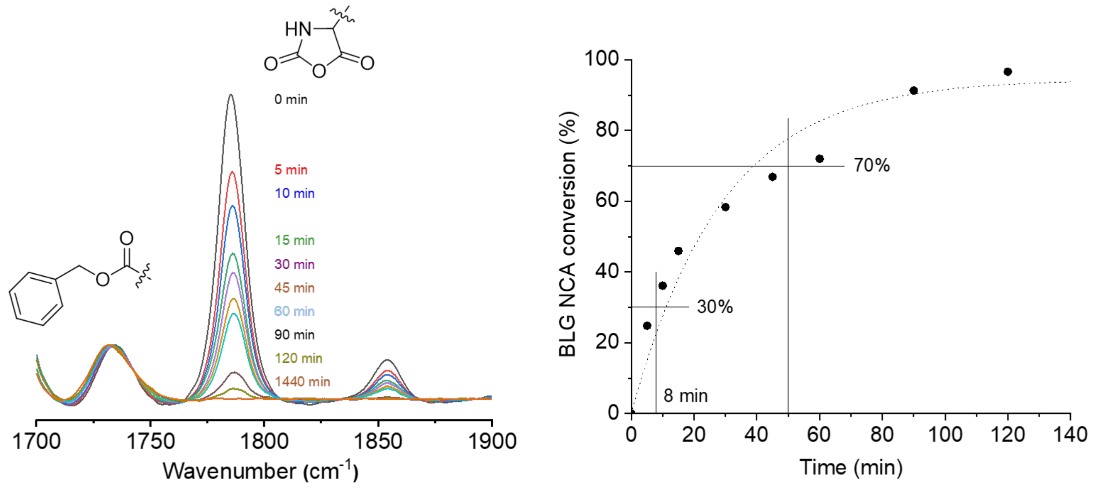

Figure S1. Kinetics of ring-opening polymerization of BLG-NCA initiated by neopentyl amine in DMF solution at room temperature, $[NCA]_0 = 1$ M, $[NCA]_0/[amine]_0 = 100$. (Left) FT-IR spectra of reaction mixture at different reaction times. (Right) Time-conversion plot.

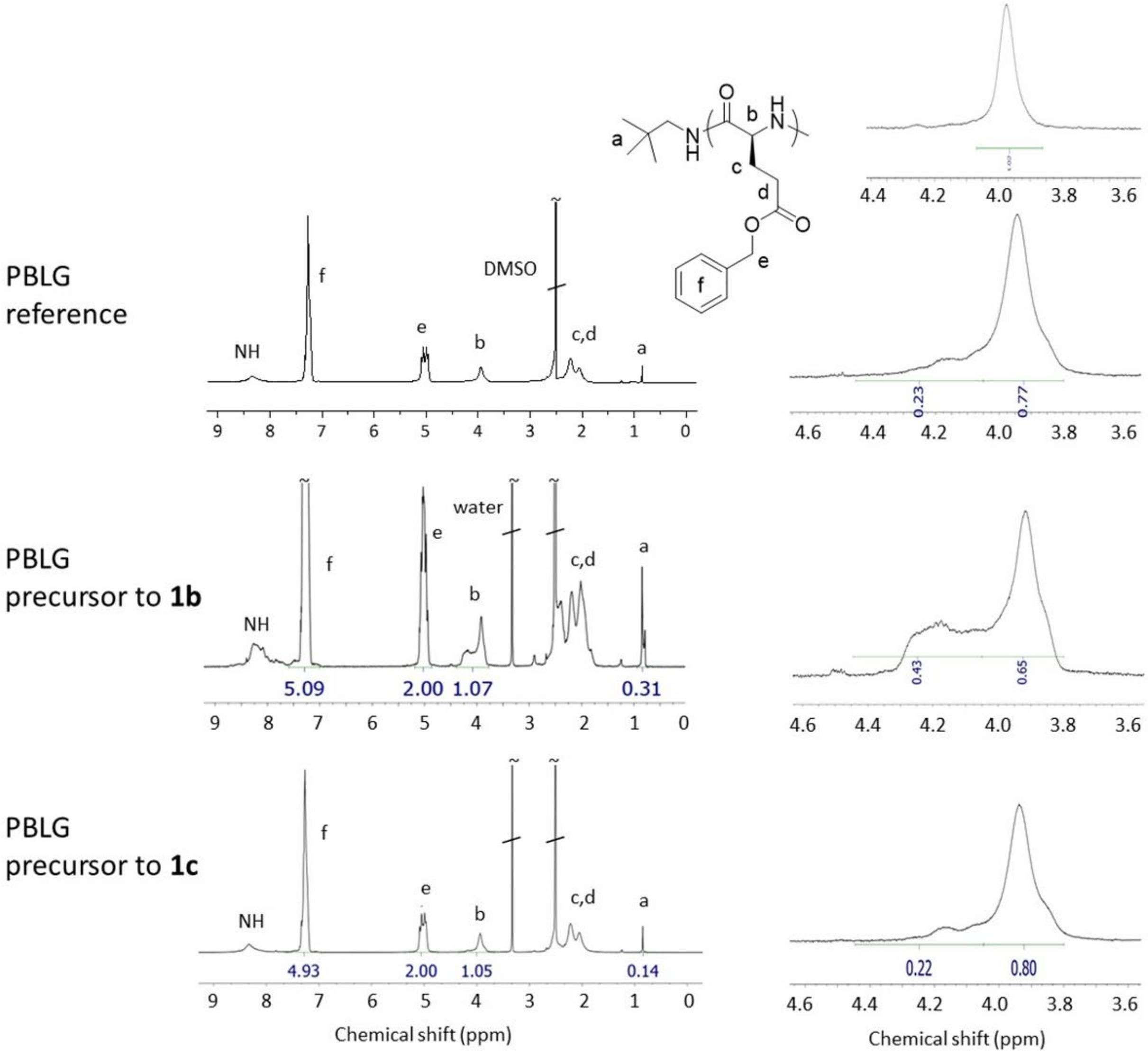

Figure S2. (Left) ^{1}H NMR spectra (400 MHz, DMSO-d_6) of PBLG (reference) and PBLG first block precursors to copolypeptides **1b** and **1c**. (Right) Expanded region of ^{1}H NMR signals of αCH protons (*b*) allowing to differentiate between α-helix (δ 3.80-4.05 ppm) and random coil (δ 4.05-4.45 ppm) conformations. Top ^{1}H NMR spectrum was recorded for PBLG (reference) in $CDCl_3$.

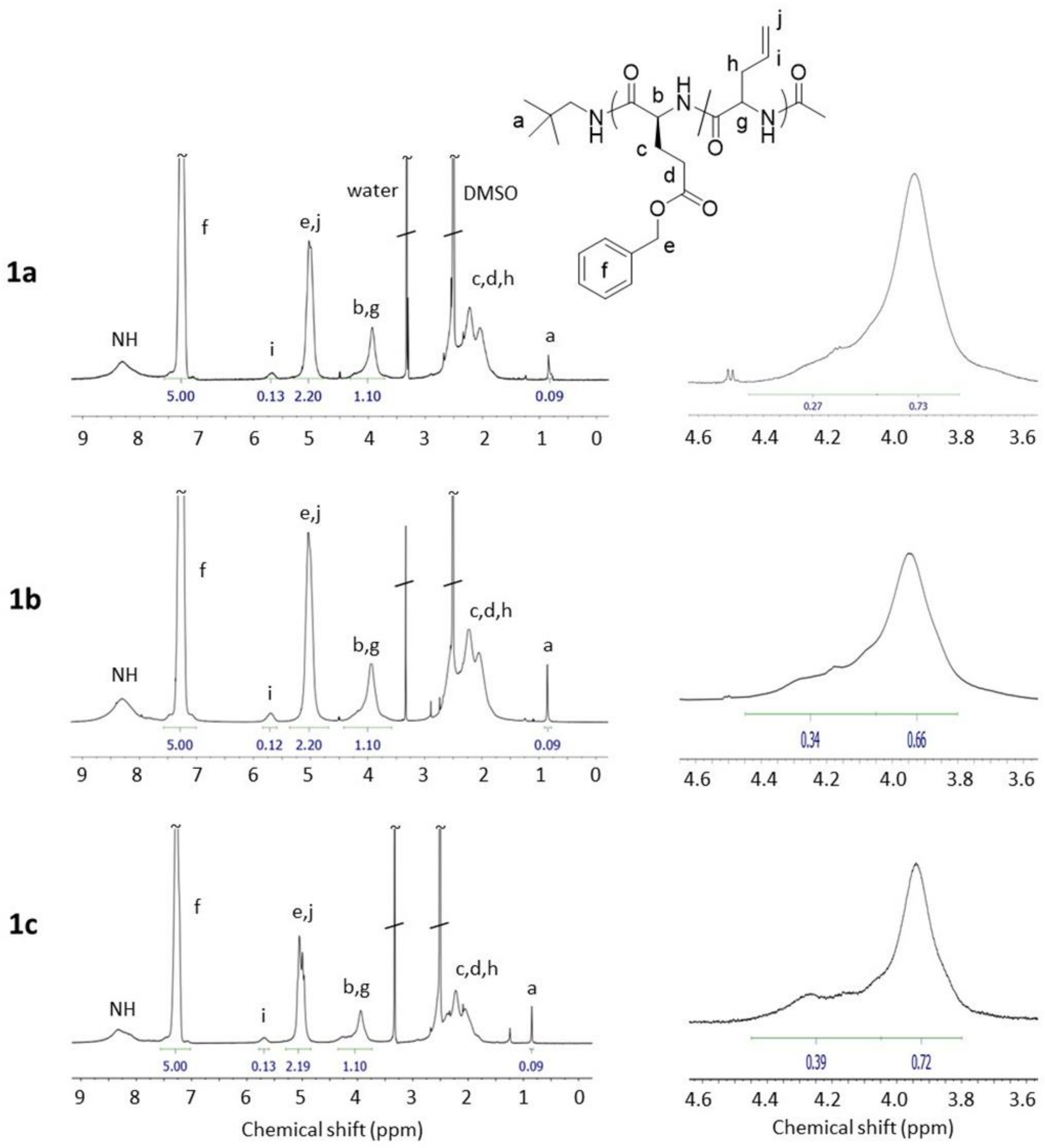

Figure S3. (Left) ^{1}H NMR spectra (400 MHz, DMSO-d_6) of the BLG/AG copolypeptides **1a-c**. (Right) Expanded region of ^{1}H NMR signals of αCH protons (*b*) allowing to differentiate between α-helix (δ 3.80-4.05 ppm) and random coil (δ 4.05-4.45 ppm) conformations.

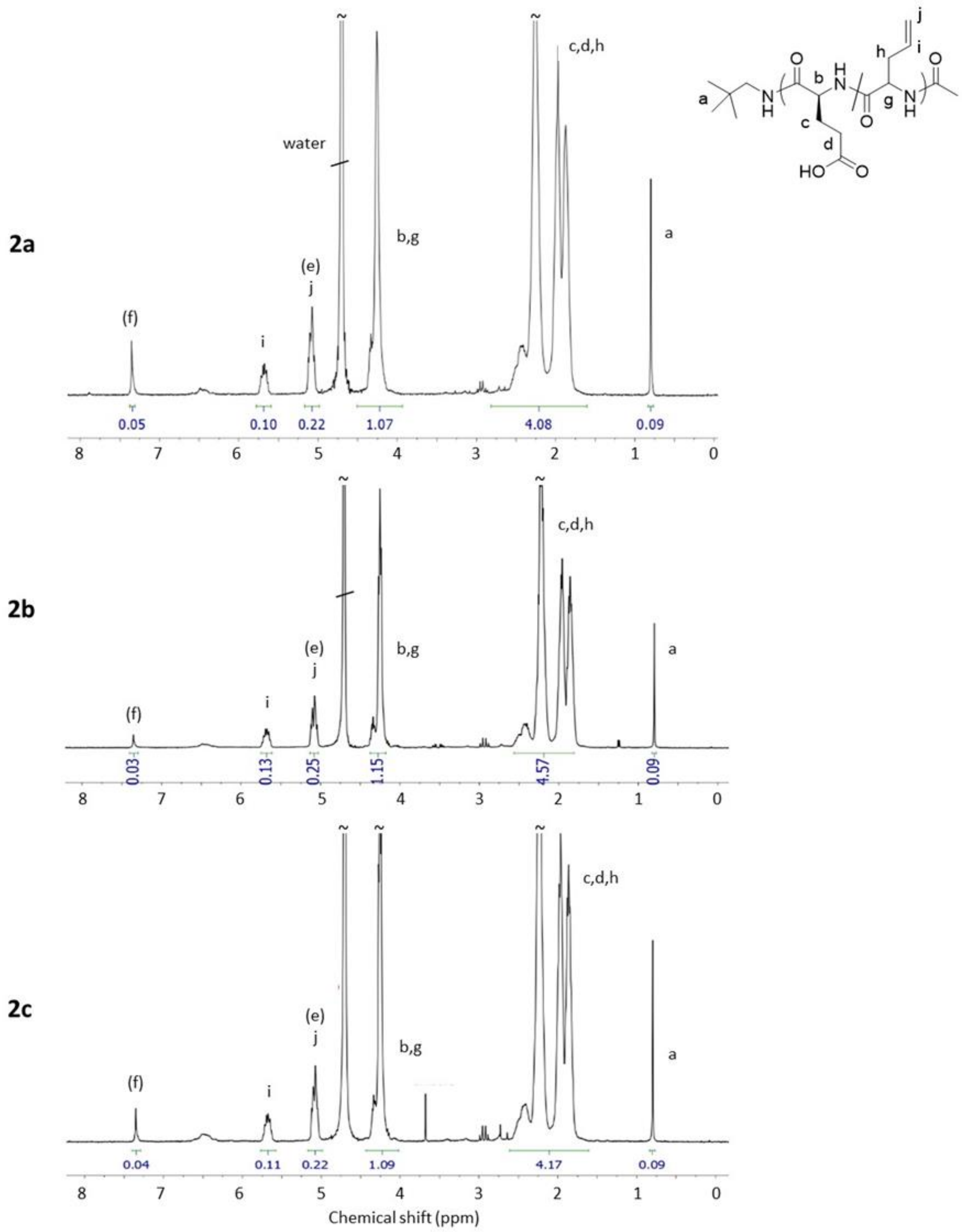

Figure S4. ^{1}H NMR spectra (400 MHz, D_2O) of the debenzylated copoly(L-glutamic acid/allyl-glycine)s **2a-c**.

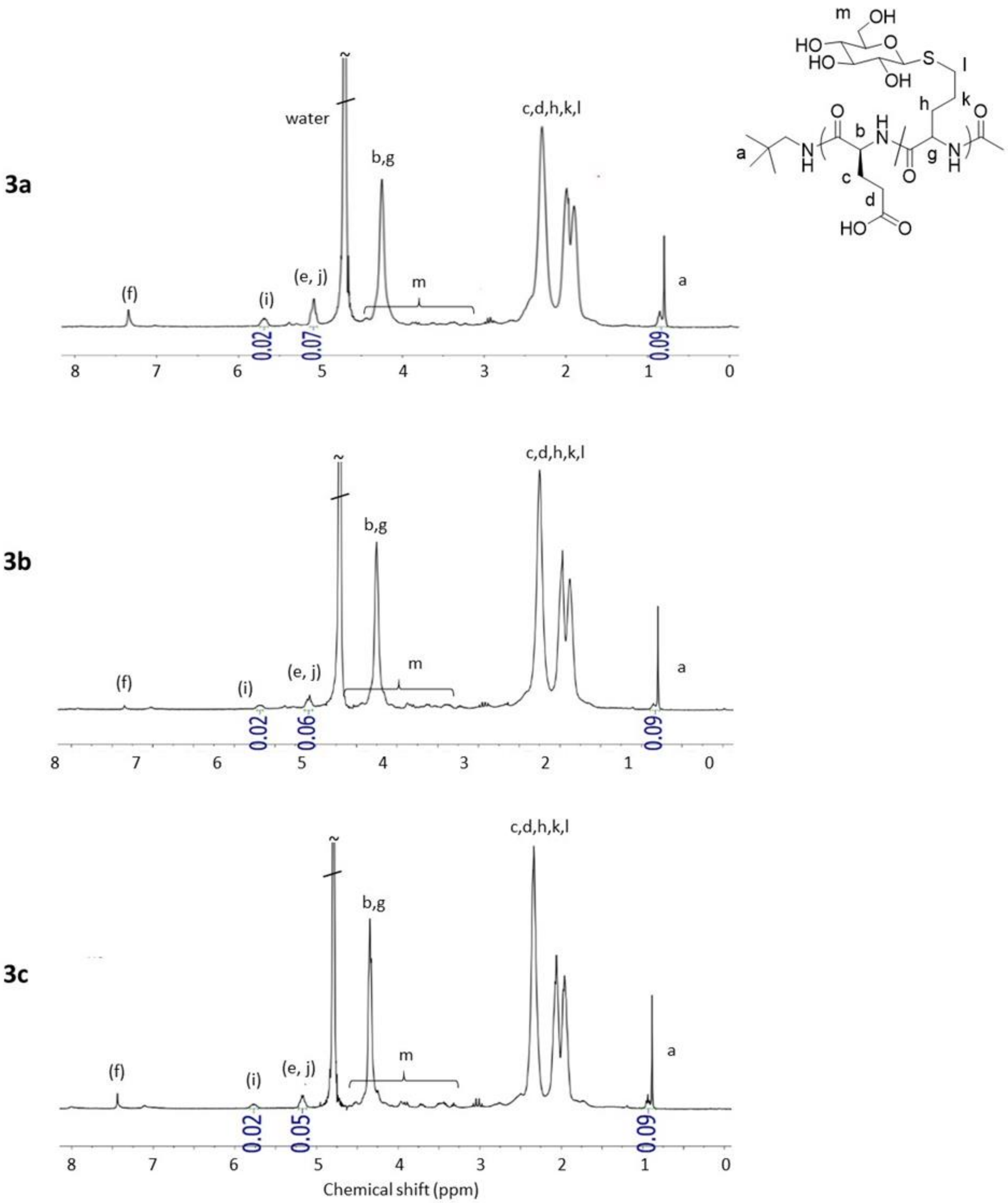

Figure S5. ^{1}H NMR spectra (400 MHz, D_2O) of the glycosylated copoly(L-glutamic acid)s **3a-c**.

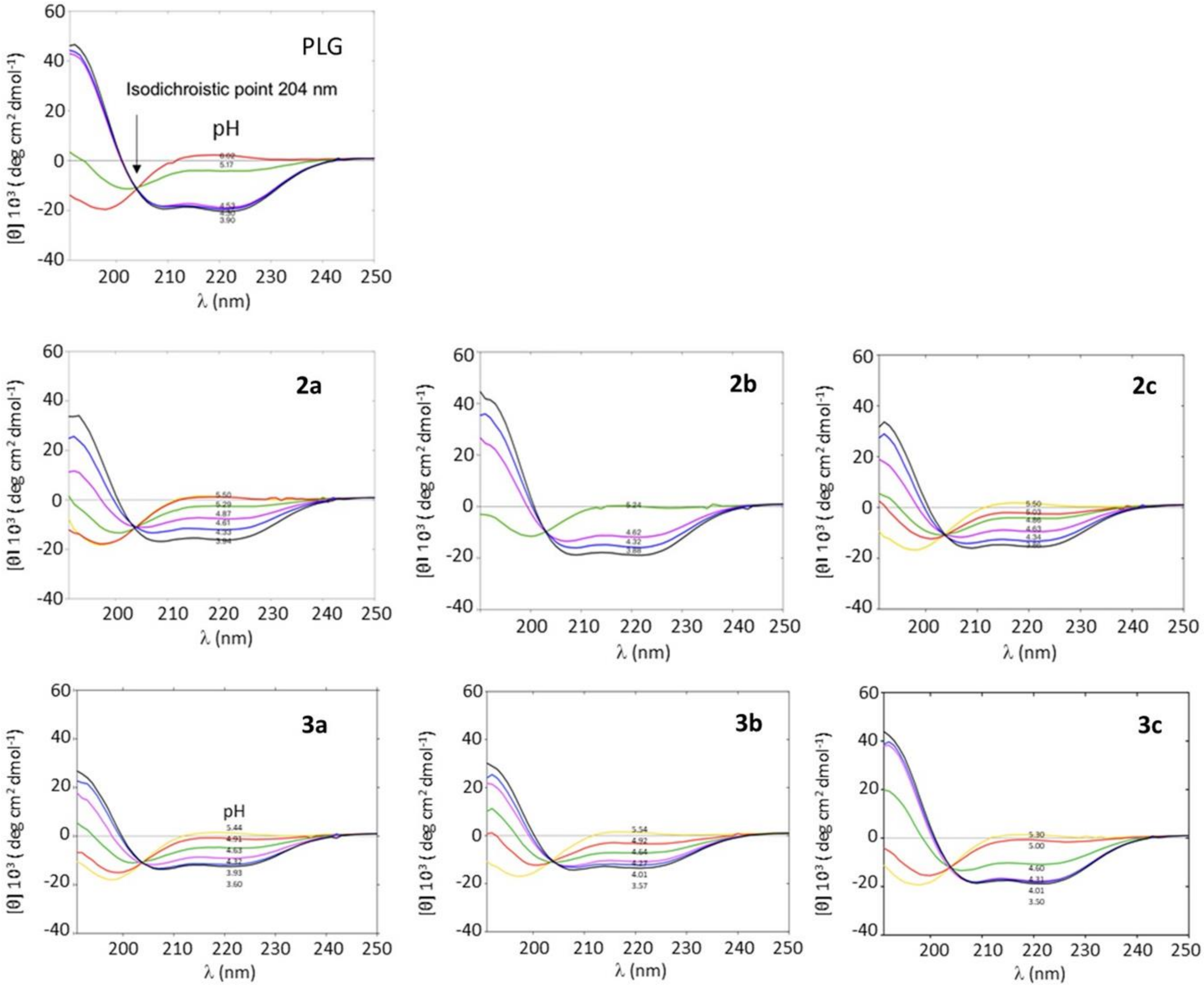

Figure S6. CD spectra of 0.1 wt% physiological saline solutions of PLG and copoly(L-glutamic acid/allylglycine)s **2a-c** (top) and glycosylated copoly(L-glutamic acid)s **3a-c** (bottom) at different pH values; room temperature.

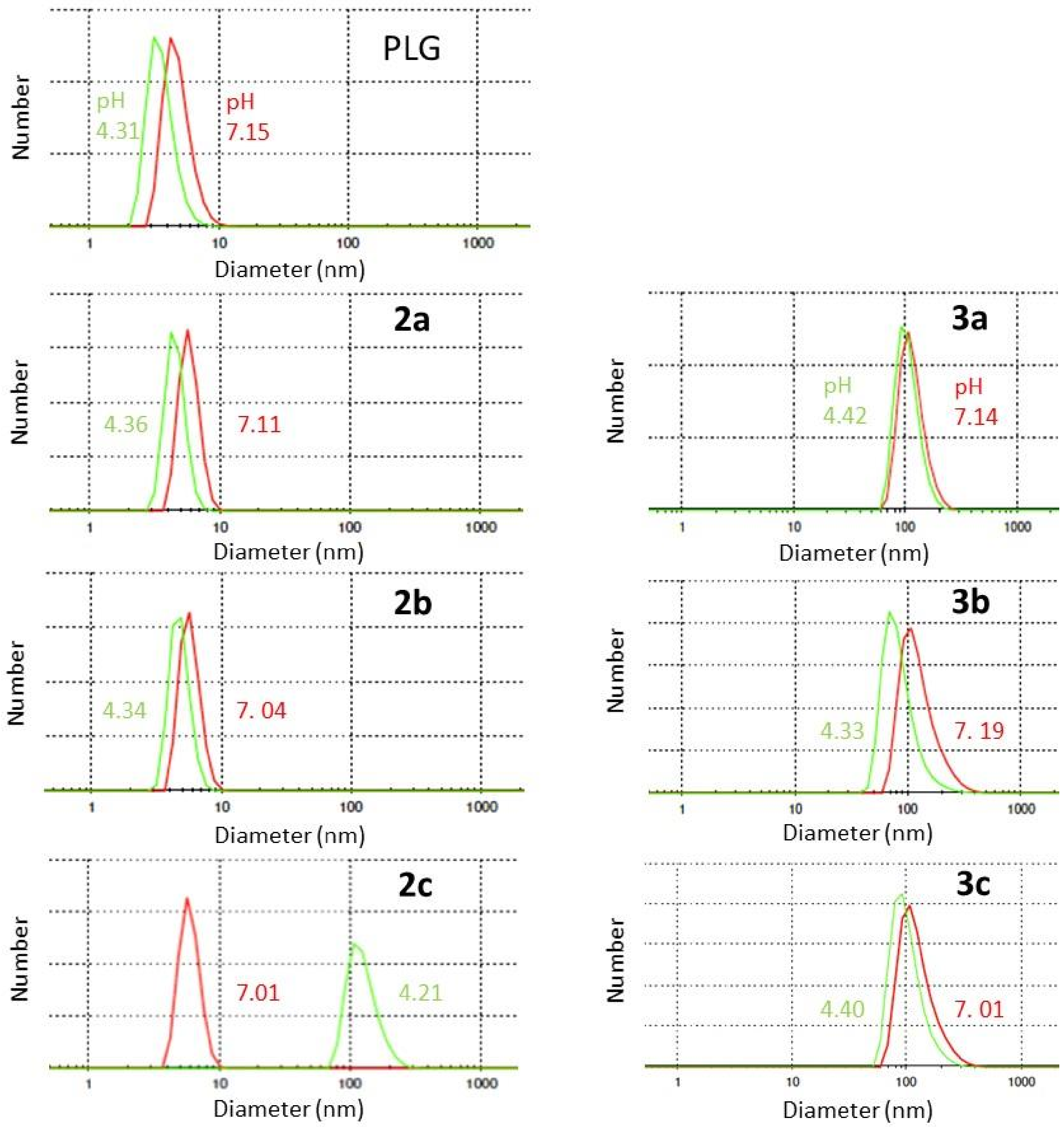

Figure S7. DLS particle size distributions by number of 0.1 wt% physiological saline solutions of PLG and copoly(L-glutamic acid/allylglycine)s **2a-c** (left) and glycosylated copoly(L-glutamic acid)s **3a-c** (right) at pH ~4.3 and pH ~7.1values; room temperature.

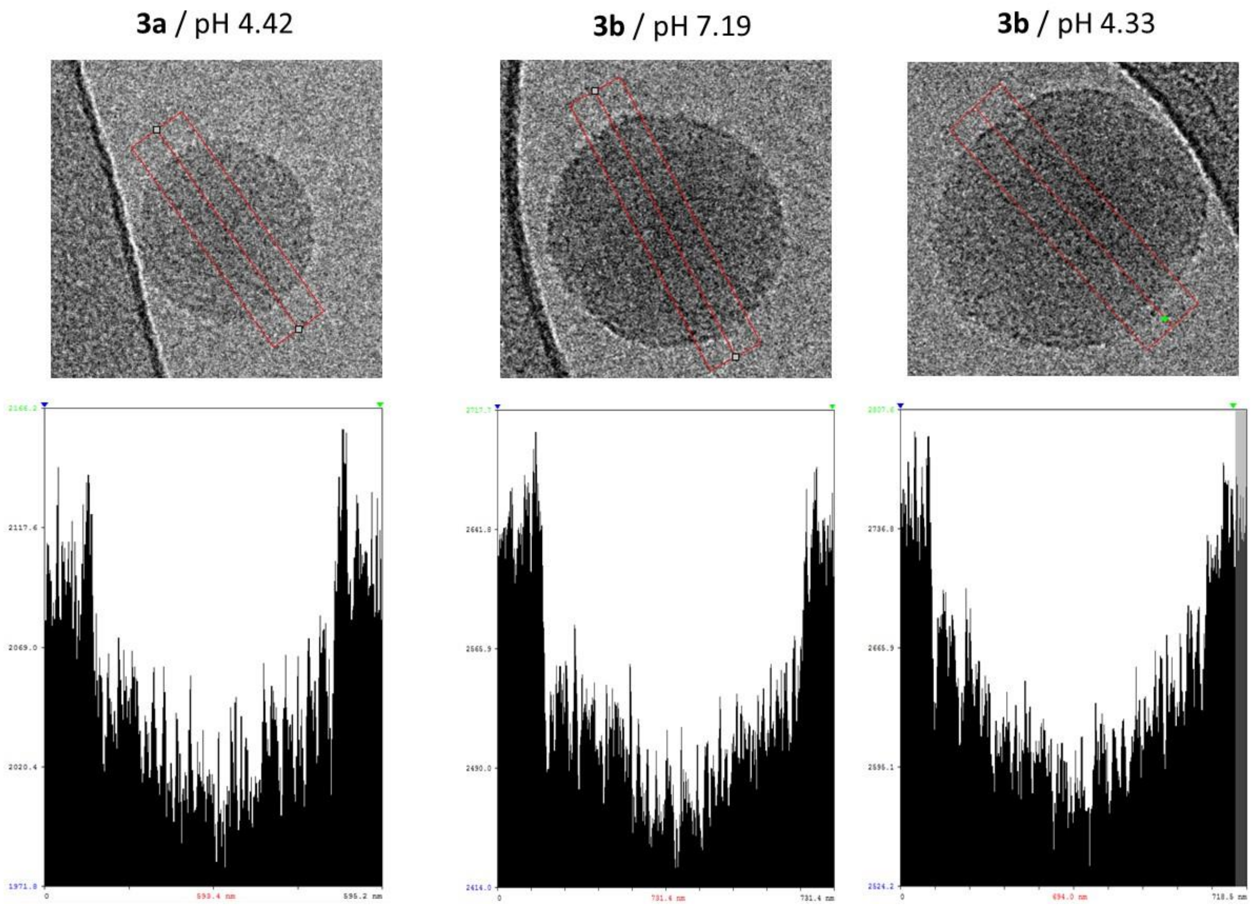

Figure S8. Cryo-TEM electron density profiles of spherical assemblies of glycosylated copoly(L-glutamic acids)s **3a** and **3b**.
